# Recent advances in understanding molecular bases of Ménière’s disease

**DOI:** 10.12703/r/12-11

**Published:** 2023-05-11

**Authors:** Lidia Frejo, Jose A Lopez-Escamez

**Affiliations:** 1 Otology & Neurotology Group CTS495, Department of Genomic Medicine, GENYO-Centre for Genomics and Oncological Research–Pfizer/University of Granada/ Junta de Andalucía, PTS, Granada, Spain; 2 Sensorineural Pathology Programme, Centro de Investigación Biomédica en Red en Enfermedades Raras, CIBERER, Madrid, Spain; 3 Department of Surgery, Instituto de Investigación Biosanitaria, ibs.Granada, Universidad de Granada, Granada, Spain; 4 Meniere's Disease Neuroscience Research Program, Faculty of Medicine & Health, School of Medical Sciences, The Kolling Institute, University of Sydney, Sydney, New South Wales, Australia

**Keywords:** Ménière’s disease, sensorineural hearing loss, otolithic membrane, tectorial membrane, endolymphatic sac, autoimmune inner ear disease, endolymphatic hydrops

## Abstract

Ménière’s disease (MD) is a rare syndromic disorder of the inner ear defined by sensorineural hearing loss (SNHL) associated with episodes of vertigo and tinnitus. The phenotype is variable, and it may be associated with other comorbidities, such as migraine, asthma, and several autoimmune disorders. The condition has a significant heritability according to epidemiological and genetic data, with a difference in comorbidities according to ethnicity. Familial MD is found in 10%, the most commonly found genes being *OTOG, MYO7A* and *TECTA*, previously associated with autosomal dominant and recessive SNHL. These findings suggest that proteins involved in the tectorial membrane and stereocilia links are critical in the pathophysiology of MD. Moreover, proinflammatory cytokines may have a role in some patients with MD by promoting a persistent inflammatory status. Preliminary data suggest that sodium intake could be related to the release of cytokines, and this may influence the relapsing course of the condition. The ionic homeostasis of the otolithic and tectorial membranes could be critical in suppressing the innate motility of individual hair cell bundles, and focal detachment of the otolithic, or tectorial membranes may cause random depolarization of hair cells and explain changes in tinnitus loudness or the triggering of vertigo attacks.

## Introduction

Ménière’s Disease (MD) is a multifactorial, chronic disorder of the inner ear, mainly defined by vertigo, low- to medium-frequency sensorineural hearing loss (SNHL), tinnitus (ringing noise in the ear), and aural fullness (feeling of pressure in the ear)^1^. The majority of patients diagnosed with MD eventually progress to chronic imbalance, moderate-to-severe SNHL in the affected ear and, frequently, persistent and disabling tinnitus. In Unilateral MD (UML), symptoms of tinnitus and SNHL develop in one ear, whereas in Bilateral MD (BMD), symptoms develop in both ears.

**Figure 1.**
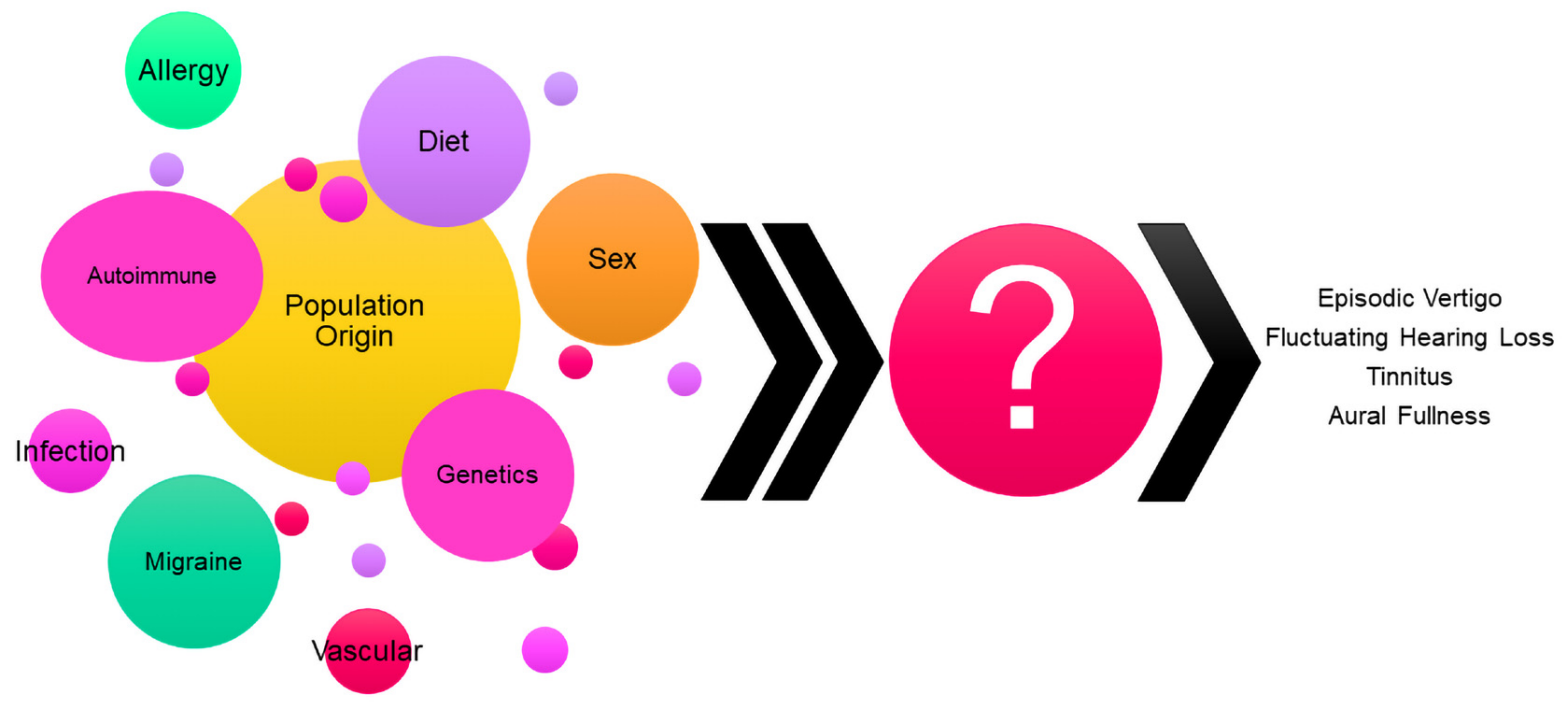
Schematic representation of factors reported to contribute to MD development.

MD is a complex and heterogeneous disorder, with multiple factors reported to contribute to its development, such as genetics, autoimmunity, allergies, migraine, sex, ethnicity, and diet, amongst others^2^ (Figure 1). Furthermore, 10 different clinical MD subgroups of patients (5 UMD and 5 BMD) have been described according to the best predictors as defining clinical subgroups with a potentially different etiology to improve the phenotyping of MD. As a result, UMD: 1) Classical MD, is the most common subgroup; 2) Delayed MD; 3) Familial MD (FMD); 4) MD with migraine; and 5) autoimmune MD; and BMD: 1) Metachronic MD; 2) Synchronic MD; 3) FMD; 4) BMD with migraine; and 5) autoimmune BMD.

Although these comorbidities are not considered in the clinical assessment, they should be in therapeutic management. Here, we introduce the last couple of years’ worth of MD research advances and propose a novel molecular hypothesis to explain episodic symptoms in MD.

### Epidemiology

Where an epidemiological study to assess a relatively rare disorder is required, adequate power and minimal sampling bias can be obtained through the use of a population-based design. The epidemiological data on MD are variable owing to changes over time in diagnostic criteria, the different methodologies used, and the populations surveyed.

Prevalence across different countries and seasonal changes seem crucial to understanding the effects of MD on social and medical aspects of different populations at present and in the future. Therefore, earlier studies estimated MD prevalence to be between 17 and 513 per 100,000 individuals^3^. What all studies seem to have in common are: 1) the prevalence of MD is higher in women than men, with reported ratios ranging from 1.3 to 4.3; and 2) the incidence increases with age, although this could be due not only to an increase in health-seeking behaviors in the older population but also to ischemic events in the inner ear mimicking MD in the elderly individuals with risk factors.

An American study comparing clinical UMD subtypes found significant differences between their cohort and the Mediterranean cohort published earlier^4^. The study included a total of 72 UMD patients. The main differences were found between UMD types 2, 3, and 5. Whilst the American cohort had greater prevalence in UMD type 2 (20.8% vs. 8%) implying a higher tendency to develop hearing loss and aural symptoms with no vertigo, both UMD type 3 and 5 were higher in the European population (FMD 4.2% vs. 13% and Autoimmune MD 4.2% vs. 11%), possibly because of the difference in sample size. It could be argued that the differences found in both studies indicate that MD might be genetically different and that environmental and epigenetic factors play important roles in defining the phenotypes in both cohorts.

In Asian countries, there had only been one epidemiologic study, conducted in Japan, which was published in 2005, where the authors used a retrospective survey of a specific district. Since then, only two studies have been published using data from the general South Korean population^2,3^. The first study revealed a swift yearly increase from 2013 to 2017 and a 2.2 female-to-male ratio. The study also analysed the potential implications of seasonal variation long-term, finding higher incidence during summer and autumn. On the other hand, the second study performed a retrospective population-based study where the authors investigated comorbidities including autoimmune, allergic, and metabolic diseases, as well as cancer and vascular risk factors^2^. The most interesting finding was the increased incidence of allergic rhinitis and allergic asthma in this cohort, who simultaneously showed a decrease in HDL cholesterol and systolic blood pressure. Furthermore, So Young Kim *et al*. also demonstrated a positive relationship between MD and prior asthma history in adults^5^. Thyroid diseases such as hypothyroidism and hyperthyroidism have been repeatedly associated with MD. In a case-control study from 2021^6^, significant evidence was found associating higher prevalence of these comorbidities to MD when compared to controls.

### Migraine

Strikingly, what all populations seem to have in common is a higher prevalence of migraine in MD patients compared to the general population. So Young Kim *et al*. has just published a case-control Korean study including a total of 6919 MD patients and 27,676 healthy controls^7^. The study revealed that 10% of MD patients had migraine compared to the 3.5% of healthy individuals, demonstrating a 2.22-fold higher risk of migraine than the matched control group. A Finnish study described an association between MD patients who experience vestibular drop attacks and migraine^8^. Another study explored the clinical and anatomical features in MD patients with and without migraine, also showing a higher prevalence of vertigo attacks in patients with MD and migraine, higher female comorbidity, poorer mastoid pneumatization, and shorter distance between the sigmoid sinus and posterior wall of the external acoustic canal^9^. A different hypothesis suggests that ionic dysregulation could play a role as a predisposing factor overlapping MD and migraine^10^.

The association between MD and migraine seems to be both an epidemiological and a mechanistic one, with up to 51% of MD individuals suffering from migraine compared to 12% in the general population. Evidence continues to emerge regarding several MD and migraine contributing factors, including spreading cortical depression, vasculopathies, calcium channelopathies, and salt intake^6^, amongst others. Furthermore, some studies suggest that rather than existing as separate conditions, MD and migraine inner ear disorder could instead exist as a continuum or have similar underlying triggers^11^.

Additionally, a study performed in a Spanish cohort of 83 patients compared two groups according to the age of onset of MD: 1) Early Onset MD (EOMD; Age of onset <35; N=44) and 2) late onset MD (LOMD; age of onset >50; N=39). They found a higher prevalence of migraine in individuals with EOMD than in patients with LOMD^12^.

### Hearing loss

The hearing profile in MD is also variable. While most patients start with a low-to-middle frequency hearing loss, some patients present a pantonal hearing loss for the first two years of the disease. This has been observed in carriers of rare variants in the *OTOG*^13^ or *TECTA*^14^ genes. Moreover, a large, multicentre study including 400 patients with UMD has found that patients with UMD and high-frequency hearing loss in the first audiogram (pantonal SNHL) have a higher risk of developing bilateral SNHL. A logistic regression model including the age of onset, the observation of high frequency hearing loss in the first audiogram, and presence of migraine can help to assess the risk of bilateral SNHL in MD^15^.

### Genetics

Most MD cases are considered sporadic, but FMD has been repeatedly described in European populations in up to 35% of cases^16^. Although there have been considerable advances in recent years, the contribution of genetic factors to MD is not yet fully understood. Historically, FMD has been predominantly described as autosomal dominant with incomplete penetrance; however, we can find great genetic heterogeneity and other types of inheritance such as recessive have been found^16^. Recently, digenic and multiallelic inheritance have also been reported in FMD^17^. This could indicate the existence of several different underlying genotypes that result in endophenotypes meeting FMD clinical criteria.

The next-generation sequencing gold standard method for the genetic diagnosis of FMD is whole-exome sequencing (WES). By that method, several rare variants and genes in different families all over the world have been described^16,18^. Intriguingly, each gene described has very different functions within the body, ranging from playing a role in the cytoskeleton structure of cochlear hair cells to axonal guidance^18^. Recently, the limbic system has also been described as participating in the pathophysiology of MD, since the gene encoding the limbic system-associated membrane protein (*LSAMP*) was described in two individuals from an Iranian autosomal recessive MD family^19^. The oxidative stress pathway has just been linked to MD predisposition by mutations in *CYP2B6* and *SLC6A* genes reported by Skarp *et al*., where they also reported candidate MD variants in *GUSB, EPB42 ASPM, KNTC1*, and *OVCH1* genes. *ASPM* and *KNTC1* variants also suggest dysregulation of the mitotic spindle formation^20^. However, the contribution of singular families to the understanding of the genetic structure of MD is low, and cellular or animal models are required to demonstrate the pathogenic effect, particularly in variants of unknown significance.

**Figure 2.**
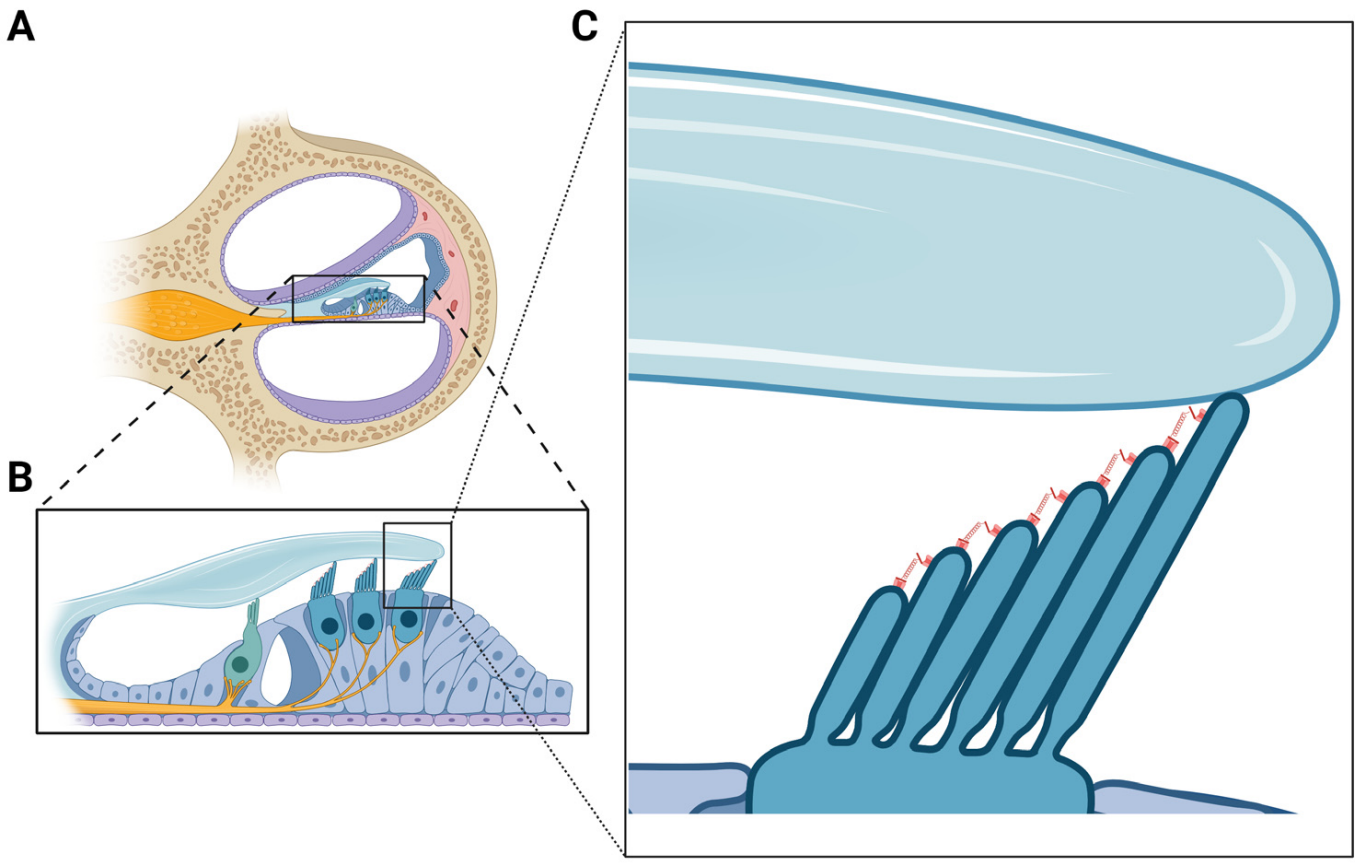
Cochlear cross-section and hair bundles. **A**. Representation of a cochlear section showing its three compartments: Scala vestibuli, Scala media, and Scala tympani. **B**. The Organ of Corti located in the Scala media with the sensory epithelia consists of inner and outer hair cells. These cells have a hair bundle in their apical surface in contact with the Tectorial membrane, an extracellular matrix of proteins that regulates the ionic composition in the tip of stereocilia. The proximate mechanical stimulus is the deflection of the hair bundles against the overlying acellular tectorial membrane. **C**. Mechanoelectrical Transduction in Hair Cells. When the hair bundle deflects toward the tallest stereocilium, cation-selective channels open near the tips of the stereocilia (tip links), allowing K^+^ to flow into the hair cell down their electrochemical gradient. The resulting depolarization of the hair cell opens voltage-gated Ca^2+^ channels, allowing calcium entry and release of neurotransmitters onto the nerve endings of the auditory nerve.

Of note, in these past couple of years, multiple MD families carrying rare variants in genes encoding proteins involved in the structure of the hair cells’ stereocilia and their attachment to the tectorial membrane (TM) have been found. Roman-Naranjo *et al*. found co-segregation in several novel and rare variants in the *MYO7A* gene with other genes like *CDH23*, *PCDH15,* and *ADGRV1* involved in the mechano-electrical transduction (MET) complex and the interciliary links of the hair cells in several MD families^21^ (Figure 2). Later, by using a Gene Burden Analysis (GBA) and applying multiallelic inheritance models in SNHL genes, enrichment of rare missense variants in the *OTOG* gene were found in 15 families with MD, suggesting multiallelic inheritance^13^. Finally, the presence of rare missense variants and frameshift deletions in the *TECTA* gene within six MD families suggests a role of this gene in the pathophysiology of the disease^14^. Otogelin is a TM protein related to secreted epithelial mucins and defects in otogelin cause a rare genetic form of deafness characterized by congenital mild-to-moderate SNHL^22^. The hair bundle proteins stereocilin, otogelin, and otogelin-like interact to form horizontal connectors between the stereocilia and the attachment of the stereocilia to the TM^23^.

Even if MD has been associated with several genes, just recently an epigenetic study was performed using whole-genome bisulfite sequencing (WGBS) suggesting that the DNA methylation signature could allow distinction between MD patients and controls^24^. In this study, the authors found a great number of differentially methylated CpGs when comparing MD patients to controls, including several previously described hearing loss genes like *CDH23*, *PCDH15,* or *ADGRV1*.

All these studies point to the proteins linking the stereocilia in hair cells of the sensory epithelia and proteins in the tectorial and otolithic membranes as molecular targets associated with the pathophysiology of FMD.

### Inflammation

Although the inner ear was considered immune-privileged for several decades, nowadays, we have plenty of evidence that this is not the case. Moreover, numerous clinical features associated with MD indicate an underlying inflammatory or autoimmune etiology.

The endolymphatic sac (ES) has been convincingly proposed as the entrance of immune cells into the inner ear, and, thus, the initiator of the immune response in this location. Histologically, it has been found that both macrophages and plasma cells reside in the perisaccular connective tissue, probably processed by the ES. Additionally, several immunological factors have been shown to be involved with the ES, such as different immunoglobulins and secretory components of the immune system.

A recent study presented the first direct measurements of cytokines in the human ES luminal fluid. Cytokines such as TNF-α, IL-6, and IFN-γ were upregulated in the ES luminal fluid in MD patients, and found to be expressed in the epithelial cells lining the sac^25^. Even though the results obtained in the ES do not correlate with those found at serum levels in the same patients, several other studies using peripheral blood mononuclear cells (PBMC) from MD patients have found an upregulation of several cytokines and chemokines. For instance, Moleon *et al*. described an upregulation of CCL18, CCL3, and CCL4 in MD patients when compared to migraine patients and healthy controls^12^.

High doses of sodium chloride (NaCl) intake have been mechanistically linked to aberrations in both the innate and adaptive immune responses. On the other hand, IL-1β has continually been shown to be over-expressed in patients with autoimmune inner ear disorder (AIED) and MD^26^. Hence, in a study performed by Pathak *et al*.^27^, they studied the effect of NaCl on cellular proinflammatory cytokine release in PBMC from MD patients, identifying that a high-salt diet triggers inflammation through the production and release of IL-1β and IL-6, resulting in the clinical exacerbation of MD.

Epigenetic changes seem to regulate cytokine profiles in MD. Part of a study mentioned above using WGBS to search for differentially methylated CpGs in mononuclear cells confirmed previous findings of a chronic inflammatory process underlying MD that could allow separate patients with different proinflammatory cytokine profiles^24^. Patients with high levels of proinflammatory cytokines have a differentially methylated region in the promoter of the IL32 gene^22^, and IL-32 can be induced by IL-1β; moreover, increased serum levels of IL-32 have been associated with various autoimmune and allergic diseases, namely type 2 diabetes, asthma, allergic rhinitis, and systemic lupus erythematosus^28^. However, the effects of IL-1β and IL-32 in the cochlea or vestibular organs are not well known.

Recent studies have demonstrated that IgE may play a role in the pathogenesis of MD. IgE, IL-4, IL-5, IL-10, and IL-13 are upregulated in some patients with MD^29^, and IL-4 can regulate the expression of CD23 in B cells and induce IgE production^30^. In vitro studies using HEI-OC1 cells have shown that CD23-mediates IgE transcytosis^29^; however, the effect of IgE and Th2 cytokines in the inner ear, including the ES deserves further investigation.

### Molecular pathophysiology leading to random activation of hair cells

The histopathological landmark of MD is the observation of dilatation of the cochlear duct secondary to the accumulation of endolymph, termed “endolymphatic hydrops” (EH). What are the mechanisms leading to this accumulation of the endolymph? Molecular genetics and inflammation research are trying to understand the molecular basis of EH.

Hair cell bundles transduce the mechanical energy of acceleration or sound pressure into electrical energy in the form of ionic currents into the sensory hair cell^31^. The otolithic membrane connects to the tops of the hair bundles and provides mechanical coupling among them. This inter-stereocilia coupling is mediated by a few extracellular proteins and it is enough to suppress the innate motility of individual bundles^32^.

The finding of rare variation leading to unstable proteins in the stereocilia or the otolithic membrane in patients with MD supports an intrinsic susceptibility in some individuals^22^ - but this is not enough, and acute environmental noise or viral infections could be triggers for the initial damage of the inner ear organs.

How can we explain the episodic nature of the condition? If the inner ear structures are damaged, then new insults, such as loud noise, could trigger acute changes in the endolymphatic pressure and cause new episodes of loud tinnitus or intense vertigo. Endolymphatic hydrops itself cannot explain episodic symptoms such as vertigo or vestibular drop-attacks. These symptoms could be better explained by focal detachment of the tectorial or otolithic membrane with loss of inhibition in spontaneous motility of hair bundles and random increase of the basal firing rate of the sensory afferents^33^.

## Summary/Conclusion

As we have seen, the past couple of years has seen significant new developments in deciphering the root causes of Ménière’s disease. As well as identifying those genes commonly associated with MD, an epigenetic component has also been elucidated. The genes shown to be involved suggest the likely involvement of proteins within the otolithic and tectorial membranes, as well as their links to stereocilia. Focal detachment of these membranes triggers spontaneous motility of hair cell bundles and random depolarization of hair cells, which may explain sudden onset of loud tinnitus or bouts of vertigo. The autoimmune aspects of the disease have also been further investigated, with the maintenance of a proinflammatory milieu in the inner ear a likely contributing factor in some patients. These developments provide us with several promising leads as potential therapeutic targets to help treat and, hopefully, eventually cure this debilitating and disabling suite of conditions.

### List of abbreviations

MD Ménière’s Disease

SNHL sensorineural hearing loss

UMD Unilateral MD

BMD Bilateral MD

FMD Familial MD

EOMD Early onset MD

LOMD Late onset MD

LSAMP Limbic system-associated membrane protein

TM Tectorial membrane

GBA Genes burden analysis

WGBS Whole-genome bisulfite sequencing

ES Endolymphatic sac

PBMC Peripheral blood mononuclear cells

AIED Autoimmune inner ear disease

EH endolymphatic hydrops
